# Hollow Bismuth Nanoparticle-Loaded Gelatin Hydrogel Regulates M2 Polarization of Macrophages to Promote Infected Wound Healing

**DOI:** 10.34133/bmr.0105

**Published:** 2024-11-11

**Authors:** Dongming Lv, Zhongye Xu, Hao Yang, Yanchao Rong, Zirui Zhao, Zhicheng Hu, Rong Yin, Rui Guo, Xiaoling Cao, Bing Tang

**Affiliations:** ^1^ Department of Burns, Wound Repair and Reconstruction, the First Affiliated Hospital of Sun Yat-sen University, Guangzhou 510080, Guangdong, China.; ^2^ Department of Dermatology, the First Affiliated Hospital of Sun Yat-sen University, Guangzhou 510080, Guangdong, China.; ^3^ Key Laboratory of Biomaterials of Guangdong Higher Education Institutes, Key Laboratory of Regenerative Medicine of Ministry of Education, Guangdong Provincial Engineering and Technological Research Center for Drug Carrier Development, Department of Biomedical Engineering, Jinan University, Guangzhou 510632, Guangdong, China.

## Abstract

Open wounds face severe bacterial infection, which affects the quality of healing. Photothermal antimicrobial therapy has received increasing attention as a broad-spectrum antimicrobial treatment that can avoid drug resistance. A variety of metallic materials have been used in the development of photothermal agents. However, there are few studies on bismuth as a photothermal agent and its use in tissue repair, so there is still a lack of clear understanding of its biomedical function. Here, a hollow bismuth nanosphere prepared from bismuth metal was developed for drug loading and photothermal antibacterial effect. The photothermal conversion efficiency of the hollow bismuth spheres reached 16.1%, and the bismuth-loaded gelatin-oxidized dextran (ODex)-based hydrogel achieves good antibacterial effects both in vivo and in vitro. The bismuth-loaded hydrogel can also promote the angiogenesis of human umbilical vein endothelial cells (HUVECs) and improve the proliferation of human keratinocytes cells (HaCaT) and the quality of wound healing. This discovery provides a new idea for the application of metal bismuth in the field of tissue repair and regeneration.

## Introduction

Bismuth (Bi) is a trace element contained in the human body and the only nontoxic heavy metal [1]. Bi pharmaceutical preparations have a long history in the field of gastrointestinal therapy. They can resist *Helicobacter pylori* infection [2], stop diarrhea, and treat dyspepsia [3]. However, research on Bi is more concentrated in the field of catalysis [4], and few studies have explored its biological role in the field of biomedicine [3]. Bi has a photothermal effect due to its high absorbance in the near-infrared (NIR) region. Most studies have focused on exploiting their intrinsic photothermal properties or developing novel nanomaterials with enhanced photothermal properties as therapeutic platforms for cancer treatment [5,6]. In addition, Bi nanomaterials also have the function of computed tomography (CT) imaging. Some studies have combined its photothermal and CT imaging functions to prepare the tumor diagnosis and treatment platform [7], but few studies have explored the role of Bi in tissue repair and regeneration.

Hydrogel can serve as a good dispersant for nanomaterials and is an advantageous material suitable for wound repair [8,9]. Hydrophilic hydrogel materials can provide a suitable environment for cell migration and growth. In addition, factors such as the material composition and mechanical strength of the hydrogel also play a crucial role in regulating the fate of cells [10]. Studies have found that methacrylic acid-modified polymer materials can regulate the polarization of macrophages from pro-inflammatory to “tissue repair” phenotypes [11–14]. Gelatin methacryloyl (GelMA) is a common degradable methacrylic acid-modified natural polymer material, and the RGD sequence contained in it can promote cell adhesion and migration [15]. Due to its excellent biocompatibility and degradability, GelMA is widely used in the field of tissue repair. GelMA mainly forms hydrogels by free radical-induced double bond polymerization, and the commonly used initiation method is photoinitiation [15–17]. Due to the abundant chemical groups in gelatin, many researchers have also used other groups to form double-crosslinked hydrogels with GelMA [18,19]. The most common one is the double-crosslinked gel formed by Schiff base reaction of amino group and aldehyde group, which is used to change the mechanical properties of the hydrogel [8,20].

Inspired by previous studies, we used GelMA and oxidized dextran (ODex) to crosslink through the Schiff base to form the hydrogel system. In order to increase the crosslinking rate and gel strength, we added gelatin into the hydrogel to increase the amino content and performed ultraviolet (UV) irradiation for secondary crosslinking. In addition to the need to prevent wound infections, another challenging issue is controlling excessive inflammatory responses at the wound site. The metabolites of hexaaminolevulinic acid hydrochloride (HAL) are carbon monoxide and bilirubin, which can effectively regulate inflammation. In order to load HAL, we prepared a photothermal agent with a hollow structure (hollow Bi nanospheres) using metallic Bi [21]. The results showed that the Bi-loaded hydrogel exhibited good photothermal antibacterial efficacy in vitro and in vivo. Furthermore, the combination of Bi and gelatin-based hydrogel effectively promoted cell migration and angiogenesis in vitro and in vivo. In the rat-infected wound models, the GO-PS/HAL@Bi hydrogel promoted the anti-inflammatory (M2) phenotype polarization of macrophages and effectively promoted wound healing.

## Materials and Methods

### Materials

Gelatin and polyvinyl pyrrolidone (PVP) were purchased from Sigma-Aldrich (Shanghai, China). Bi nitrate pentahydrate and methacrylic anhydride were purchased from Macklin (Shanghai, China). Sodium periodate and ethylene glycol were purchased from Aladdin (Shanghai, China). CCK-8 kit was purchased from Beyotime (Shanghai, China). Live/dead staining kit and rhodamine B-labeled phalloidin were purchased from Keygen (Jiangsu, China). Mannitol sodium chloride selective medium was purchased from Huankai Microbial (Guangzhou, China). Gram-negative bacteria selective medium was purchased from Hopebiol (Qingdao, China). CD206 polyclonal antibody and inducible nitric oxide synthase (iNOS) polyclonal antibody were purchased from Proteintech (Wuhan, China). Dulbecco’s modified Eagle’s medium (DMEM), fetal bovine serum (FBS), penicillin–streptomycin, and trypsin were purchased from Gibco (Shanghai, China). All reagents were used without any purification.

### Synthesis and chemical structure characterization of GelMA and ODex

GelMA and ODex was synthesized according to previous report [8,9]. Briefly, gelatin was dissolved in phosphate-buffered saline (PBS), and then methacrylic anhydride was added to gelatin solution and reacted for 1 h. The solution was dialyzed against deionized water and lyophilized to obtain GelMA [9]. Dextran was dissolved in deionized water and reacted with sodium periodate. Ethylene glycol was used to consume excess sodium periodate, and the solution was dialyzed against deionized water and lyophilized to obtain ODex [8]. The obtained GelMA and ODex were dissolved in deuterated water (D_2_O), and hydrogen spectra were determined by a nuclear magnetic resonance (NMR) spectrometer.

### Synthesis and characterization of hollow Bi nanoparticles and HAL@Bi nanoparticles

#### Synthesis of Bi and HAL@Bi nanoparticles

Bi nitrate pentahydrate (0.182 g) was dissolved in 5 ml of 1 M nitric acid. PVP (0.15 g) was dissolved in 25 ml of ethylene glycol and added to the Bi nitrate solution. The above solution was transferred to hydrothermal reactor and reacted at 150 °C for 12 h. The mixture was centrifuged at 10,000 rpm for 15 min to obtain the precipitate and washed with deionized water for 3 times. Precipitation was freeze-dried to obtain hollow Bi nanoparticles. The prepared hollow Bi was characterized by x-ray diffraction analysis (XRD), nano-laser particle size analysis, and transmittance electronic microscopy (TEM).

For preparing HAL@Bi nanoparticles, the HAL solution (8 mg/ml) was stirred with of hollow Bi (2 mg/ml) at a volume ratio of 1:1 for 24 h in ice bath, centrifuged at 10,000 rpm for 15 min to obtain the precipitate, and washed with deionized water for 3 times.

#### Drug loading, encapsulation efficiency, and in vitro drug release of hollow Bi spheres

HAL loading into hollow Bi spheres was performed by mixing a hollow Bi dispersion (10 ml, 1 mg/ml) with HAL (10 mg) in deionized water. After stirring for 24 h at 4 °C, HAL@Bi was obtained through high-speed centrifugation at 10,000 rpm for 10 min and washed in deionized water for 3 times. The standard curve of HAL was derived from UV–visible (Vis) spectrum (265 nm). The drug loading capacity and encapsulation efficiency were calculated using the following equation:

$$\begin{array}{c} \begin{array}{c} \text{Drug loading capacity}\;\left(\%\right)=\\ \frac{\text{weight of devoted drug}\;-\;\text{weight of drug in supernatant}}{\text{weight of hollow bismuth}}\times 100\% \end{array} \end{array}$$

$$\begin{array}{c} \begin{array}{c} \text{Encapsulation efficiency}\;\left(\%\right)=\\ \frac{\text{weight of devoted drug}\;-\;\text{weight of drug in supernatant}}{\text{weight of devoted drug}}\times 100\% \end{array} \end{array}$$

The release of HAL from hollow Bi spheres and hydrogels was monitored by measuring the drug concentration in the release medium. The GO-PS/Bi hydrogel or hollow Bi spheres were placed into cellulose dialysis bags, which were then immersed in the release medium. At specified time intervals, aliquots of the release medium were withdrawn and replaced with an equal volume of fresh medium to maintain consistent conditions. The NIR-responsive release of HAL was initiated by exposing the HAL@Bi to NIR laser irradiation at a power density of 1.5 W/cm^2^ for 5 min.

### Preparation and characterization of gelatin-based hydrogel

#### Preparation of hydrogel

The gelatin-based hydrogel was prepared following Table, in which GO-P means GelMA and ODex crosslinked by photo-initiation; GO-S means GelMA and ODex crosslinked by Schiff base; GO-PS means GelMA and ODex double crosslinked by photo-initiation and Schiff base. The photo-initiated hydrogel was formed by 405-nm light irradiation for 1 min.

**Table.** Construction of gelatin-based hydrogel

	GelMA	ODex	Gelatin	Bi	HAL@Bi	LAP
GO-P	10%	5%	/	/	/	0.2%
GO-S	/	5%	10%	/	/	/
GO-PS	5%	5%	5%	/	/	0.2%
GO-PS/Bi	5%	5%	5%	0.2 mg/ml	/	0.2%
GO-PS/Bi@HAL	5%	5%	5%	/	0.2 mg/ml	0.2%

LAP, lithium phenyl-2,4,6-trimethylbenzoylphosphinate

#### Gelation time of hydrogel before light irradiation

The gelation time of the gelatin-based hydrogel before light irradiation was measured with a rotational rheometer (Kinexus Pro, Malvern) at 37 °C. The hydrogel prepared following Table was immediately added to the machine, and the moduli changing with time were tested within fixed strain (1%). The gel point is the intersection of storage modulus (*G*′) and loss modulus (*G*″). Gelation time was then recorded by the gel point.

#### Compression tests of hydrogel

Cylindrical hydrogel samples were prepared according to Table. Universal testing machine was used to test the compressive strength of the hydrogel at a steady speed (0.05 mm/s). The compressive force (*F*) and displacement were recorded, and the compressive strength was calculated following the formula:

Compressivestrength(Pa)=F/S

where *S* represents the cross-sectional area of the hydrogel sample.

### In vitro photothermal activity of hollow Bi nanoparticles and GO-PS/Bi hydrogel

The photothermal activity of hollow Bi was measured using the following procedures. Hollow Bi nanoparticles were ultrasonically dispersed in deionized water at concentrations of 50, 100, 200, 300, 400, and 500 μg/ml. Hollow Bi suspension (1 ml) was the irradiated with 808-nm NIR laser for 5 min at a power density of 2.0 W/cm^2^. Temperature was recorded every 20 s by a thermocouple thermometer. Hollow Bi suspension at 200 μg/ml was irradiated with an 808-nm NIR laser at different power densities from 0.8, 1.0, and 1.5 to 2.0 W/cm^2^ for 5 min. Temperature was recorded every 20 s by a thermocouple thermometer. The photothermal stability was investigated using an on–off laser cycle for 5 times. Hollow Bi suspension (1 ml) at 200 μg/ml was irradiated with 808-nm NIR laser for 5 min at a power density of 1.5 W/cm^2^, and then the laser was turned off until the temperature of suspension went back to room temperature. The process was continued for 5 times. The light-to-heat conversion efficiency was calculated according to the method reported in the literature [22,23].

The photothermal degradation property of hollow Bi suspension was detected by a UV–Vis spectrophotometer, and 5 min was an illumination cycle. The absorbance of hollow Bi suspension at 400 to 900 nm was detected by a UV–Vis spectrophotometer immediately after NIR laser irradiation. A hollow Bi suspension without NIR laser irradiation was used as control.

The GO-PS, GO-PS/Bi, and GO-PS/HAL@Bi hydrogels were irradiated with 808-nm NIR laser for 5 min at a power density of 1.5 W/cm^2^ for 5 min, and thermal images were obtained with an infrared thermal camera every 1 min. Water with NIR laser irradiation was used as control.

### Evaluation of antibacterial activity in vitro

The GO-PS-based hydrogel (200 μl) was prepared in a 24 well-plate and sterilized under UV light (365 nm) for 6 h. One milliliter of *Escherichia coli* or *Staphylococcus aureus* bacterial suspensions (1 × 10^7^ CFU ml^−1^) dispersed in lysogeny broth (LB) liquid culture medium was added to the surface of the hydrogel, and the hydrogel was then treated with or without 808-nm NIR light at a power density of 1.5 W/cm^2^ for 5 min. Sterilized PBS buffer (200 μl) was used as the control group. Then, the well plate was cultured at 37 °C for 4 h. Finally, the number of bacteria was determined by the spread plate method.

### In vitro cytotoxicity analysis and cytoskeleton staining

#### Cytotoxicity analysis of hollow Bi

HaCaT cells were seeded on the 96-well plates with a concentration of 10,000 cells per well and cultured for 24 h. Hollow Bi nanoparticles were ultrasonically dispersed in DMEM complete culture medium (supplemented with 10% FBS and 1% penicillin–streptomycin) at concentrations of 50, 100, 200, 300, 400, and 500 μg/ml. Then, the culture medium was replaced with the hollow Bi dispersion and cultured for another 24 h. After culturing, the culture medium was carefully washed by PBS buffer, and cell viability was then tested by the CCK-8 method according to instructions provided by the manufacturer.

#### Cytotoxicity analysis of hydrogel

Hydrogel extracts were prepared according to a previous method [20]. In brief, hydrogels were immersed in DMEM complete culture medium with a mass ratio of 1:10 at 37 °C for 24 h to obtain hydrogel extracts. Human umbilical vein endothelial cells (HUVECs) and human keratinocytes cells (HaCaT) cultured with DMEM complete culture medium were digested, resuspended (100,000/ml), and seeded in 96-well plates (100 μl per well). After culturing for 24 h to allow the cells to adhere to the wall of the well plate, the culture medium was replaced with the hydrogel extract to continue culturing for 24 h. Cell viability was then tested the by CCK-8 method according to instructions provided by the manufacturer.

#### Live/dead staining and cytoskeleton staining of HUVECs and HaCaT cells

HUVECs and HaCaT cells were seeded in 24-well plates (1 × 10^5^ cells per well). Cells were cultured in DMEM complete culture medium for 24 h and then replaced by hydrogel extracts for another 3-d culturing. For live/dead staining, cells were washed with PBS and then stained with Calcein-AM/PI live/dead staining kit according to instructions provided by the manufacturer. For cytoskeleton staining, cells were first fixed with 4% paraformaldehyde and then stained following the instructions provided by the manufacturer.

### In vitro cell migration of HUVECs

The scratch wound assay was used to evaluate the cell migration effect of Bi-containing hydrogels on HUVECs. The HUVECs were seeded in 24-well plates (1 × 10^5^ cells per well) and cultured in DMEM complete culture medium to form monolayer of confluent cells. Then, a scratch was made in each well using a pipette tip (20 μl) and cells were cultured with the hydrogel extract or DMEM basal medium (control). Photos of scratches were captured with an inverted microscope.

### In vitro tube formation

Matrigel (100 μl) was added in a 48-well plate and incubated at 37 °C for 30 min to form a gel. Then, HUVECs (100,000 per well) were seeded on Matrigel and cultured with the hydrogel extract for 4 h. Photos of tubes formed by HUVECs were captured with an inverted microscope, and the number of junctions and meshes was calculated by ImageJ software with the angiogenesis analyzer plugin.

### In vitro polarization of RAW 264.7 cells

RAW 264.7 cells seeded on 6-well plates were first polarized by LPS (100 ng/ml) for 24 h and then cultured with hydrogel extracts for 24 h. To analyze phenotypic switching of RAW 264.7, they were analyzed by flow cytometry and immunofluorescence. M1-type RAW 264.7 cells were labeled with iNOS antibody, and M2-type RAW 264.7 cells were labeled with CD206 antibody.

### In vivo infected wound healing model establishment and treatment

Sprague–Dawley (SD) rats were randomly divided into 5 groups (*n* = 6). After the rats were anesthetized, the hair on the back was removed, and the skin with a diameter of 12 mm was cut off on the back with ophthalmic scissors. Four wounds were formed on the back of each rat. One hundred microliters of *E. coli* and *S. aureus* bacterial suspension [1 × 10^8^ colony-forming units (CFUs)/ml] was injected on each wound, closed by Tegaderm, and infected for 24 h. After bacterial infection, the hydrogel was injected on the wound surface via a syringe. For groups requiring NIR laser irradiation, hydrogels were irradiated with a power density of 1.5 W/cm^2^ for 5 min. After 3, 7, 12, and 19 d of treatment, the rats were euthanized, and the back skin was obtained, fixed with 4% paraformaldehyde for more than 24 hours, sectioned, and stained to evaluate the healing quality.

The number of wound bacteria was counted using the skin on the 3rd day. Skin (0.1 g) was harvested and homogenized in 1 ml of sterilized saline. Then, the tissue homogenate was diluted with sterile saline and spread on agar plates (*n* = 6). The plate was incubated upside down at 37 °C for 24 h, and then the number of colonies was calculated.

### Statistical analysis

All data were presented as means ± standard deviation, and at least 3 parallel samples were contained in each group. GraphPad Prism 7.0 software was used to analyze the significant difference. Differences among groups were evaluated using one-way analysis of variance (ANOVA), followed by the Tukey test when performing multiple comparisons between groups. The statistical significance was *P < 0.05, **P < 0.01, and ***P < 0.001.

## Results and Discussion

### Structure and in vitro photothermal characterization of hollow Bi

**Fig. 1.**
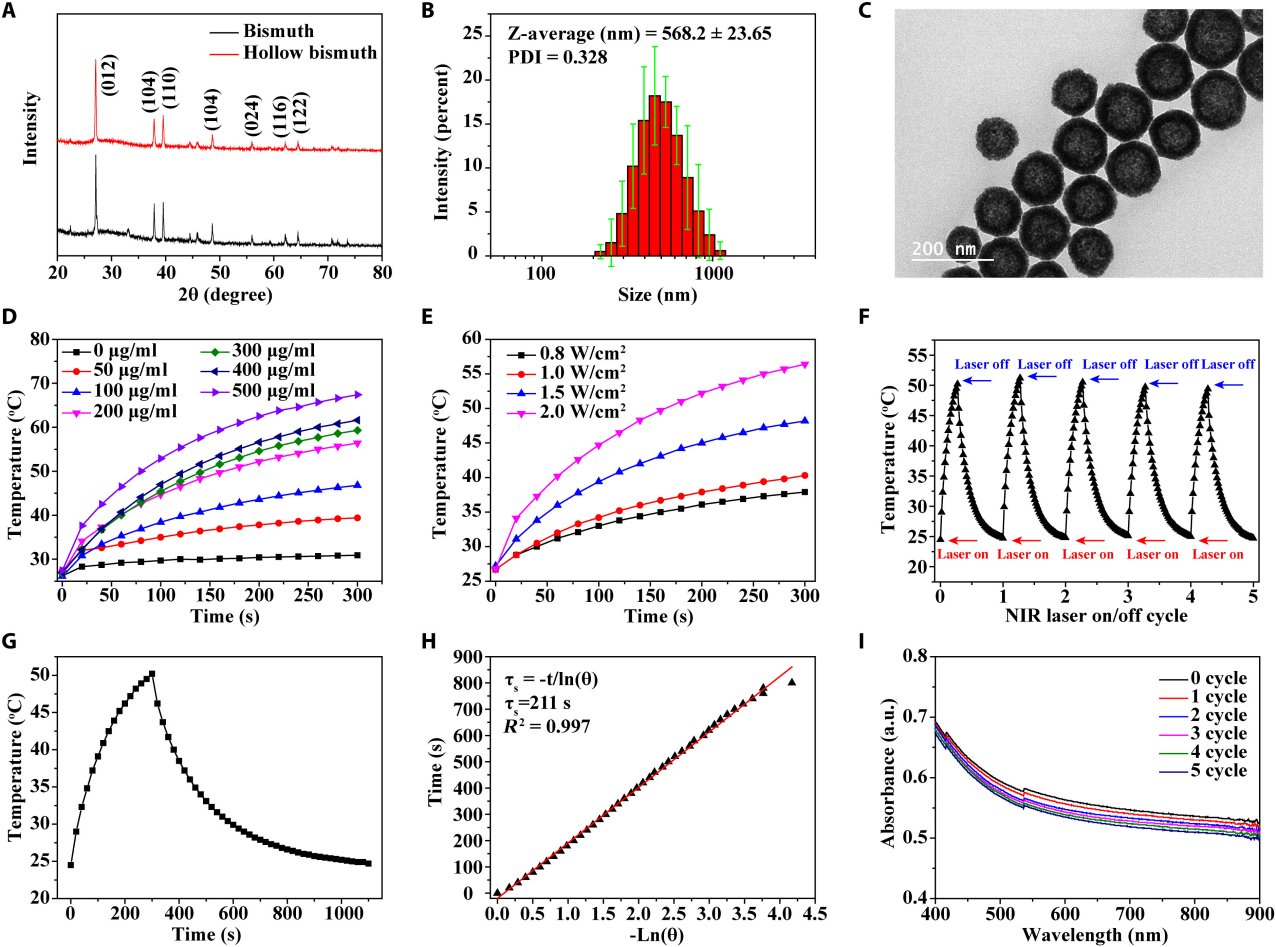
Structure and in vitro photothermal ability of the hollow Bi. (A) XRD patterns of Bi and hollow Bi. (B) Diameter dispersion of hollow Bi in deionized water measured by DLS. (C) TEM image of the hollow Bi. (D) Heating curves of hollow Bi with different mass concentrations (0, 50, 100, 200, 300, 400, and 500 μg/ml) irradiated with 808-nm NIR laser for 5 min at a power density of 2.0 W/cm^2^. (E) Heating curves of hollow Bi at 200 μg/ml irradiated with 808-nm NIR laser at different power densities (0.8, 1.0, and 1.5 to 2.0 W/cm^2^) for 5 min. (F) On–off cycling heating curves of hollow Bi at 200 μg/ml irradiated with 808-nm NIR laser (1.5 W/cm^2^) for 5 min on each cycle. (G) Single on–off heating curve of hollow Bi at 200 μg/ml irradiated with 808-nm NIR laser (1.5 W/cm^2^) for 5 min. (H) Time constant for heat transfer from the system is determined to be τ_s_ = 211 s by applying the linear time data from the cooling period (after 300 s) versus the negative natural logarithm of the driving force temperature, which is obtained from the cooling stage of (G). (I) UV–Vis spectra of hollow Bi (200 μg/ml) after irradiating for 5 cycles.

We prepared Bi nanospheres with hollow structures for loading HAL by hydrothermal reaction. First, the crystal phase of hollow Bi nanospheres was characterized, and the XRD spectrum (Fig. 1A) showed that the peak positions of the powdered Bi and the prepared hollow Bi nanospheres were consistent. These peaks were the crystal faces of the rhombohedral phase of Bi, proving that the prepared hollow Bi spheres did not change the structure of Bi, and the prepared hollow Bi spheres were not oxidized [24,25]. The particle size distribution and morphology of the hollow Bi spheres were subsequently characterized by dynamic light scattering (DLS) and TEM. The hydrated particle size of the hollow Bi spheres was uniformly distributed around 568 ± 23.65 nm (Fig. 1B), and the TEM image clearly showed that the middle part of the nanospheres is lighter in color, forming a hollow spherical structure (Fig. 1C). Bi has strong absorption in the NIR region, which leads to its photothermal properties [26]. We explored the photothermal effect of hollow Bi spheres in vitro and found that the heating temperature and concentration are positively correlated under the NIR light irradiation of the fixed power (2.0 W/cm^2^) (Fig. 1D). The temperature variation amplitude varies significantly between 0 and 200 μg/ml of hollow Bi spheres. When the Bi sphere concentration is higher than 200 μg/ml, the temperature rise tends to be gentle. The cytotoxicity of hollow Bi at different concentration has also been evaluated on HUVEC and HaCaT cells, and the results were shown in Figs. S1 and S2. The cell viability of HUVEC began to decline significantly above 300 μg/ml, but it could still reach 81.6% at 300 μg/ml; for HaCaT cells, the cell viability was higher than that of the control group when the concentration was lower than 400 μg/ml. We chose 200 μg/ml of hollow Bi spheres as the final concentration to balance the photothermal performance requirements and the cytotoxicity of HUVEC and HaCaT cells. Then, the heating temperature under different powers was measured. When the laser power density was 1.5 W/cm^2^, the highest temperature of the hollow Bi sphere solution was 48.2 °C after irradiating for 5 min. The highest temperature reached 56.4 °C when the power density was 2.0 W/cm^2^, while the temperature was lower than 40 °C when the power density was lower than 1.5 W/cm^2^ (Fig. 1E). In order to achieve the photothermal antibacterial effect and avoid damage to normal tissues caused by excessive local temperature (>50 °C), we finally added 200 μg/ml hollow Bi spheres and irradiated them under NIR light of 1.5 W/cm^2^ for 5 min to achieve the photothermal antibacterial effect. The hollow Bi sphere solution still maintained a stable photothermal heating effect after 5 photothermal cycles (Fig. 1F). After extracting and calculating the data of the heating period and the cooling period, the time constant of the heat transfer of the system was τ_s_ = 211 s and the final calculated photothermal conversion efficiency was 16.1% (Fig. 1G and H). We detected the UV–Vis light absorbance of the solution after each photothermal cycle at 400 to 900 nm and found that the absorbance of the solution of hollow Bi spheres after photothermal treatment gradually decreased (Fig. 1I), while the absorbance of the untreated solution was basically unchanged (Fig. S3). The photothermal effect led to a certain degree of oxidation of the outer layer of the hollow Bi sphere, so the absorbance gradually decreased. When the Bi is oxidized to Bi oxide, the photothermal ability will be lost [27,28]. The prepared hollow Bi spheres can withstand multiple photothermal cycles.

### Characterization of physical and chemical properties of materials

**Fig. 2.**
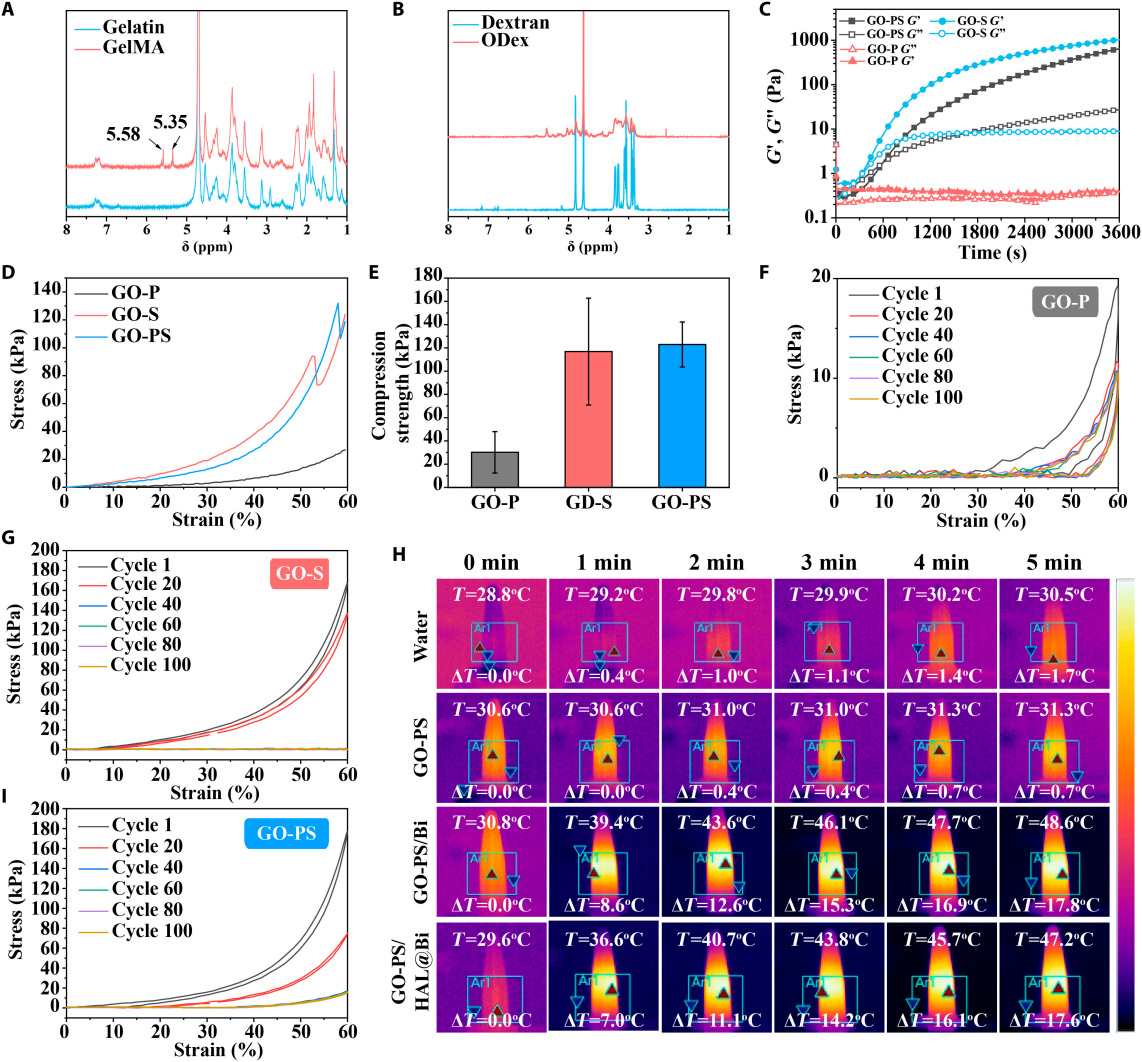
Chemical structures and physiochemical properties of GO-PS hydrogel. (A) ^1^H NMR spectra of gelatin and GelMA. (B) ^1^H NMR spectra of dextran and ODex. (C) Time sweep sequence of GO-P, GO-S, and GO-PS hydrogel. (D) Strain–stress curves of GO-P, GO-S, and GO-PS hydrogel. (E) Maximum compression strength of GO-P, GO-S, and GO-PS hydrogel before fracture at 60% strain. Circular strain–stress curves of (F) GO-P, (G) GO-S, and (I) GO-PS hydrogel. (H) Thermal images of water, GO-PS, GO-PS/Bi, and GO-PS/HAL@Bi irradiated with 808-nm NIR laser (1.5 W/cm^2^) for 5 min.

The hydrogel is obtained by double crosslinking of gelatin, GelMA, and ODex through photo-crosslinking and Schiff base reaction [20]. The chemical structures of GelMA and ODex were characterized by ^1^H-NMR spectroscopy. The chemical shifts of the active hydrogens of the methylene groups in the methacryloyl group in GelMA appeared at 5.58 and 5.35 ppm (Fig. 2A), and the chemical shifts of ODex were located at 4.3 to 5.6 ppm (Fig. 2B) [20]. In previous studies, GelMA and ODex have been used to prepare double-crosslinked hydrogels for wound repair, but the mechanical strength of hydrogels formed by the combination of 5% GelMA and 5% ODex was low (<30 kPa) [8,20], because most of the amino groups in gelatin are substituted by methacryloyl groups, and the number of amino groups that can form Schiff base groups with aldehyde groups is low. To enhance the mechanical strength of the hydrogel, we additionally added 5% gelatin to increase the density of Schiff base crosslinks. As shown in Fig. 2C, only GelMA and ODex (GO-P) were difficult to form hydrogels through Schiff base crosslinking at the same mass fraction and there was no obvious modulus change after mixing for 1 h. At the same ODex content, the gelation rate was significantly accelerated after adding 5% GelMA and 5% gelatin (GO-PS). The GO-S hydrogel formed a gel in 5 min, and their *G*′ and *G*″ were basically parallel after 20 min, indicating that the content of amino groups significantly affects the crosslinking rate of the Schiff base and gel strength. We performed mechanical strength tests on GO-P, GO-S, and GO-PS hydrogels after secondary crosslinking induced by irradiation. As shown in Fig. 2D and E, the Schiff base crosslinking hardly enhanced the strength of the hydrogel due to the low amino group content of the GO-P hydrogel. The compressive strength of the GO-PS hydrogel obtained by replacing 5% GelMA with 5% gelatin was significantly enhanced. Compared with GO-S, the maximum compressive strength of the GO-PS hydrogel was around 120 kPa, which was nearly 100 kPa higher than that of the GO-P hydrogel. The results of hydrogel cyclic compression showed that the GO-P hydrogel had better circulatory performance, while its strength was the lowest (Fig. 2F). In addition, when used as the wound dressing, the GO-P hydrogel still needs additional photo-crosslinking equipment to form the hydrogel, which increases the complexity of use. Both GO-S and GO-PS hydrogels were difficult to withstand more than 20 cycles of compression, which is due to the difficulty of slipping between molecular chains due to the high crosslinking density of the hydrogels. The reformation of Schiff base bonds took a certain time, so the hydrogel was easily broken under the rapid external force (Fig. 2G and I). The advantage of GelMA in the GO-PS hydrogel is that when the hydrogel needs to be repaired quickly, it can be triggered by providing external light, thereby reducing the healing time of the hydrogel (the hydrogel is quickly formed by light triggered within 30 s).

We finally chose the GO-PS hydrogel as the base material of wound dressing for loading the prepared hollow Bi spheres. Figure 2H shows the photothermal effect based on the GO-PS hydrogel. The hydrogel itself had no photothermal effect. But when hollow Bi spheres were added, the hydrogel had excellent photothermal performance and could rapidly increase the temperature to 17.8 °C, making the hydrogel meet the needs of antibacterial applications by destroying bacterial structures through photothermal effects [29]. Moreover, the photothermal properties of the hydrogel were not affected after the hollow Bi spheres were loaded with HAL.

### Characterization of photothermal antibacterial behavior in vitro

**Fig. 3.**
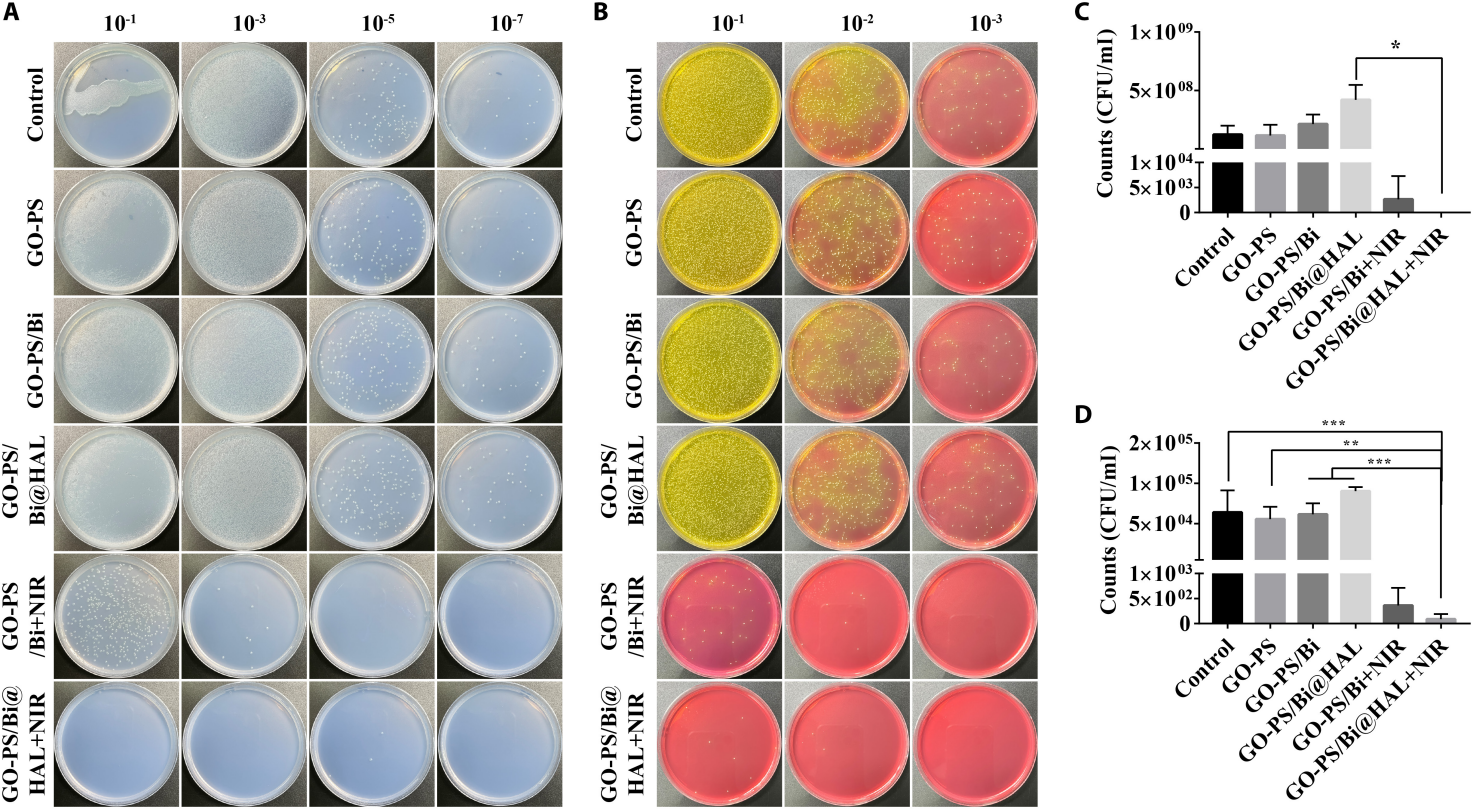
In vitro antibacterial activity of GO-PS-based hydrogel with/without NIR laser irradiation. Images of agar plates of (A) *E. coli* and (B) *S. aureus* after dilution at different times, and related colony counts of (C) *E. coli* and (D) *S. aureus* (*n* = 4, *P < 0.05, **P < 0.01, ***P < 0.001).

GO-PS/Bi and GO-PS/HAL@Bi hydrogels prepared based on GO-PS have excellent photothermal properties. We used this photothermal property to achieve the bactericidal effect and verified it through in vitro experiments. Figure 3A and B shows the killing effect of the hydrogel on *E. coli* and *S. aureus*, respectively. The GO-PS/Bi and GO-PS/HAL@Bi hydrogels exhibited excellent killing effects on both bacteria when irradiated by NIR laser. The GO-PS/Bi and GO-PS/HAL@Bi hydrogels almost completely inhibited the growth of bacterial colonies under NIR irradiation, indicating that the photothermal effect based on hollow Bi spheres can provide hydrogels with excellent antibacterial effects. These results suggest that the GO-PS/Bi and GO-PS/HAL@Bi hydrogels can be used for anti-infection wound healing application in vivo.

### Bi improves the angiogenesis and cell migration of HUVECs

**Fig. 4.**
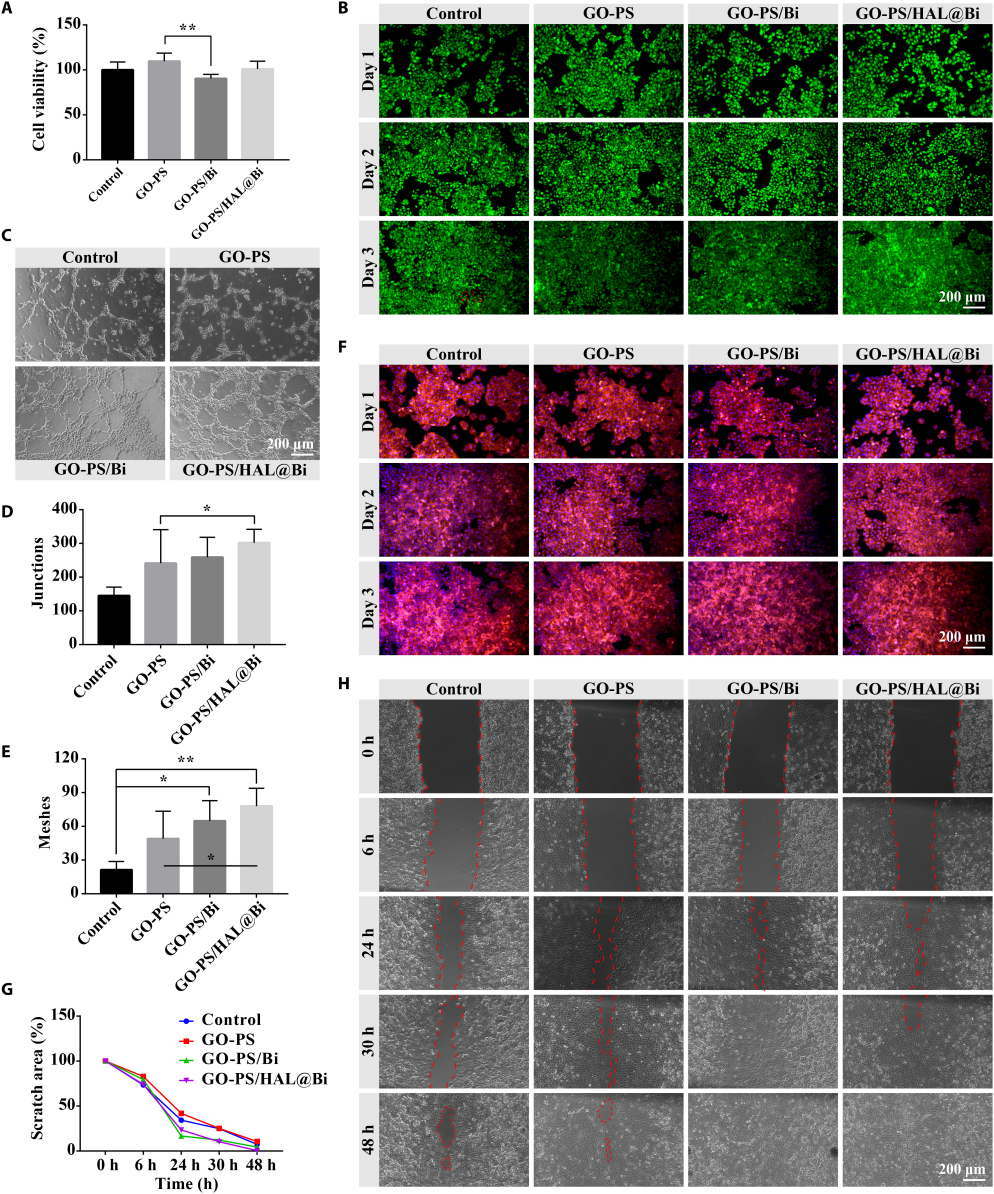
Evaluation of the effect of GO-PS/HAL@Bi hydrogel on HUVECs. (A) Cell viability of HUVECs treated with hydrogel extract for 24 h detected by the CCK-8 method (*n* = 4, *P < 0.05, **P < 0.01). (B) Live/dead staining of HUVECS treated with hydrogel extract for 24 h. (C) Images of in vitro tube formation of HUVECs. (D) Junctions and (E) meshes formed in the tube networks (*n* = 4, *P < 0.05, **P < 0.01). (F) Cytoskeleton staining of HUVECS treated with hydrogel extract for 24 h. (G) Statistical values and (H) images of scratch area (*n* = 4).

The accelerated rate of vascularization significantly enhances wound healing [30,31]. We used HUVECs to study the effect of Bi-containing hydrogels on angiogenesis effect in vitro. The cytotoxicity of the hydrogel was first explored. Although the addition of Bi decreased the cell viability of HUVECs, the overall cell viability values were >80%, indicating that the GO-PS/Bi and GO-PS/HAL@Bi hydrogels had no cytotoxicity (Fig. 4A). HUVECs were subsequently stained for live/dead fluorescence and cytoskeleton. The number of live cells labeled with green fluorescence gradually increased over time. The number of red fluorescent-labeled dead cells gradually increased after 3 d of culture, which was caused by the increase in cell density (Fig. 4B). Intracellular F-actin was labeled in red to show the cytoskeleton. The cytoskeleton is concerned with the anchor links between cells. The tighter the links between cells, the higher the mechanical strength of the resulting cellular ensemble. Figure 4F shows that HUVECs treated with the hydrogel did not affect the connection between cells and could form the tightly connected whole after 2 to 3 d of culture, which made the new tissue densely packed during tissue regeneration parts.

The effect of the hydrogel on angiogenic behavior was shown in Fig. 4C, where HUVECs cultured on Matrigel treated with the GO-PS/HAL@Bi hydrogel formed the most obvious vascular network. In addition, the GO-PS/Bi hydrogel also had the effect of promoting angiogenesis on Matrigel (Fig. 4C). After counting the number of network junctions and meshes formed by HUVECs, we found that the statistical values of both were significantly higher in the Bi-containing hydrogel group than in the control group (Fig. 4D and E). We also investigated whether the Bi-containing hydrogel could promote the migration of HUVECs by observing changes in the scratch area at different time points through scratch assays (Fig. 4H) and performing quantitative statistics on the area change values (Fig. 4G). The scratch wounds in the Bi-containing hydrogels groups almost disappeared after 30 h of culture, and the migration rate of HUVECs was relatively faster than that of the group without Bi. This is the first time that Bi has a certain promoting effect on cell migration and angiogenesis of HUVECs.

### Bi promotes the proliferation of HaCaT cells and regulates the polarization of RAW 264.7 cells

**Fig. 5.**
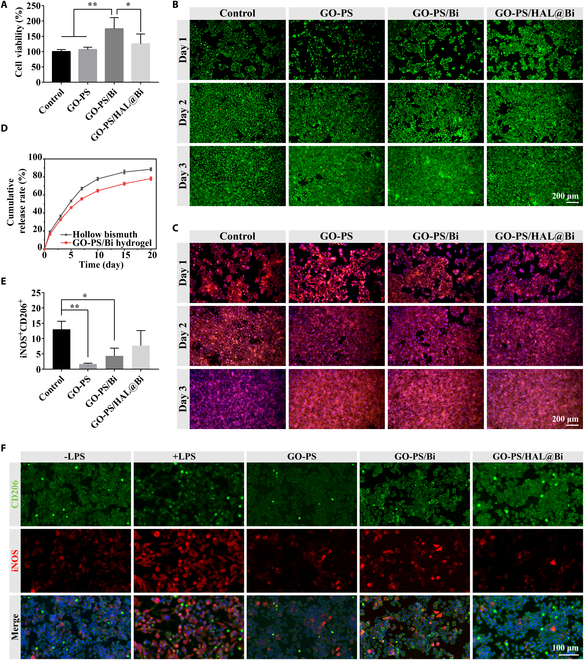
Evaluation of the effect of GO-PS/HAL@Bi hydrogel on HaCaT cells and RAW 264.7 cells. (A) Cell viability of HaCaT cells treated with hydrogel extract for 24 h detected by the CCK-8 method (*n* = 4, *P < 0.05, **P < 0.01). (B) Live/dead staining of HaCaT cells treated with hydrogel extract for 24 h. (C) Cytoskeleton staining of HaCaT cells treated with hydrogel extract for 24 h. (D) Cumulative release rate curves of HAL from hollow Bi and GO-PS/HAL@Bi hydrogel. (E) Quantitative data of iNOS and CD206 expression obtained by flow cytometry analysis of LPS-induced RAW 264.7 cells treated with GO-PS, GO-PS/Bi, or GO-PS/HAL@Bi (*n* = 3, *P < 0.05). (F) Representative immunofluorescence images of LPS-induced RAW 264.7 treated with GO-PS, GO-PS/Bi, or GO-PS/HAL@Bi hydrogel.

HaCaT cells and macrophages are also 2 main types of cells involved in the wound healing process [32,33]. HaCaT cells are involved in the formation of the epidermis during wound healing. We also studied the effect of the hydrogel on the cell viability of HaCaT cells. GO-PS/Bi significantly improved the cell viability of HaCaT cells by more than 150%, while the GO-PS group was almost unchanged compared with the control group, which indicated that the addition of Bi had a significant effect on improving the viability of HaCaT cells (Fig. 5A). Then, we also studied the effect of Bi addition on HaCaT cells by cell live/dead staining and cytoskeleton staining. The results of live/dead staining of HaCaT cells on day 3 showed that the density of living cells labeled with green fluorescence was higher in the Bi presence group than in the control group (Fig. 5B). Cytoskeleton staining also showed that the hydrogel can promote the formation of the cytoskeleton, strengthen the link between cells, and form a tighter whole, which can be observed in HaCaT cells treated with the hydrogel with or without Bi. The results indicated that the main function of Bi was to improve the cell viability of HaCaT cells, and the GO-PS hydrogel could promote the formation of the cytoskeleton of HaCaT cells due to the presence of gelatin. The presence of Bi has different effects on different cells, and the HUVEC cells explored in the previous section did not change significantly after being cultured with Bi.

The purpose of HAL as an immunomodulator is to participate in the metabolic formation of intracellular bilirubin and carbon monoxide (CO) and regulate the polarization of macrophages from M1 to M2 phenotype. Wu et al. [21] used M2 macrophage-derived exosomes to load HAL by electroporation for the treatment of atherosclerosis. Inspired by this study, we loaded HAL with hollow Bi spheres in an attempt to achieve a similar effect. The drug loading and encapsulation efficiency of HAL in hollow Bi spheres were determined to be 24.51 ± 2.71%. Under NIR light stimulation, HAL was more effectively released from the hollow Bi spheres through photothermal effects (Fig. S4). Over the course of a 20-d release experiment, the cumulative release of HAL from the hollow Bi spheres reached 88.56 ± 1.65%, whereas the release from the GO-PS/Bi hydrogels was 78.16 ± 1.92%. The release rate of HAL from the GO-PS/Bi hydrogels was lower than that from the hollow Bi spheres, which is attributed to the hydrogels’ stronger sustained-release effect by encapsulating the HAL@Bi nanoparticles within the hydrogel matrix (Fig. 5D).

RAW 264.7 is polarized toward the M1 phenotype upon induction by lipopolysaccharide (LPS). M1-type RAW 264.7 cells were treated with hydrogels, and flow cytometry was used to label iNOS and CD206 on the macrophage surface to determine their phenotype. It was found that the number of M1-type macrophages treated with hydrogels was lower after LPS induction compared to the untreated control group (Fig. S5). Quantitative analysis of flow cytometry results verified that GO-PS-based hydrogels can significantly reduce the number of M1 macrophages, and the GO-PS/HAL@Bi hydrogel can increase the number of M2 macrophages (Fig. 5E). iNOS and CD206 were then labeled by immunofluorescence staining. Uninduced RAW 264.7 cells were observed to be round with weak iNOS expression and strong CD206 expression. Cells induced by LPS were found to be spindle-shaped, with increased iNOS expression. After LPS-induced RAW 264.7 cells were treated with GO-PS-based hydrogels, iNOS expression was attenuated, and CD206 expression in cells treated with GO-PS/Bi and GO-PS/HAL@Bi was higher than in those treated with GO-PS alone (Fig. 5F). One of the main components of the GO-PS hydrogel is GelMA. Research has found that methacrylic acid material (MMA) is capable of polarizing macrophages into the “pro-regenerative” phenotype through an insulin-like growth factor 1 (IGF-1)-mediated pathway. Carleton and colleagues [11,14] increased the expression of pro-regenerative M2 macrophage markers by implanting degradable hydrogels containing methacrylate group (MAA) in vivo. According to these results, it can be inferred that the GO-PS hydrogel we prepared has a certain effect on macrophage phenotype regulation, and the addition of Bi and HAL can endow the hydrogel with antibacterial, cell proliferation, and angiogenesis effects.

### NIR light triggers the antibacterial effect of GO-PS/HAL@Bi hydrogel and promotes wound healing

**Fig. 6.**
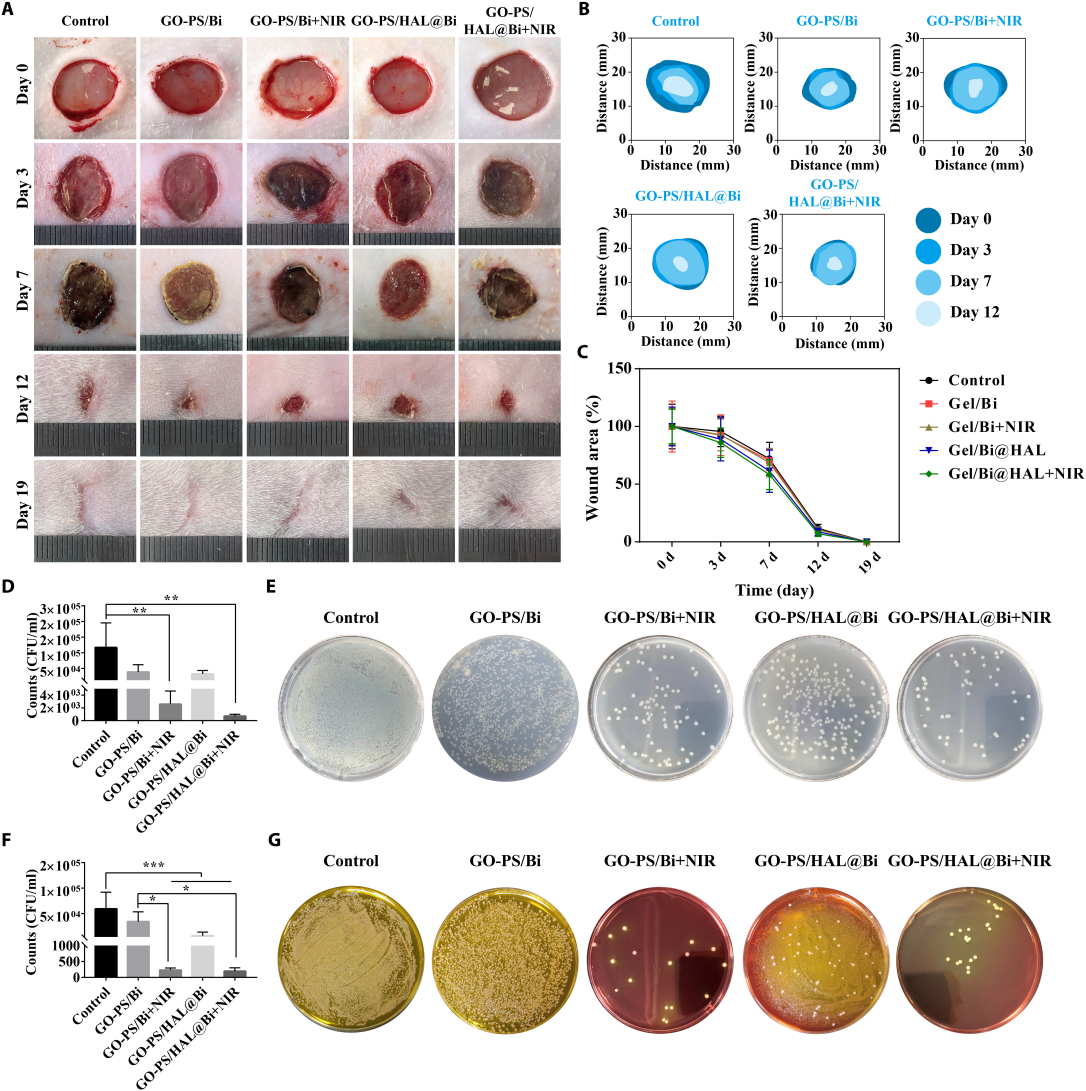
Healing rate of infected wound and evaluation of antibacterial effect in vivo. (A) Representative wound images of SD rats treated with different hydrogels at different times. (B) Images of wound area changes in different groups. (C) Wound area change curves of SD rats treated with different methods. (D) Count of *E. coli* in vivo and (E) images of colonies after 10-fold dilution in tissue fluid (*n* = 3, *P < 0.05, **P < 0.01, ***P < 0.001). (F) Count of *S. aureus* in vivo and (G) images of colonies after 10-fold dilution in tissue fluid (*n* = 3, *P < 0.05, **P < 0.01, ***P < 0.001).

After infected wounds were treated with the hydrogel, the healing rate did not differ significantly between the groups. However, the wounds treated with GO-PS/Bi + NIR and GO-PS/HAL@Bi + NIR scabbed more completely, formed a complete protective layer by the 3rd day, and exhibited no exudate around the scab by the 7th day. In contrast, the control group showed more exudate due to infection (Fig. 6A to C). The photothermal effect of Bi was triggered by the NIR laser, which makes the local temperature rise to exert the antibacterial effect. After rat wounds were treated with the GO-PS/Bi and GO-PS/HAL@Bi hydrogels and irradiated at a power density of 1.5 W/cm^2^ for 5 min, the skin temperature at the injured site rose to 50.8 °C, reaching the temperature at which bacteria are killed (Fig. S6). The number of bacteria at the wound site was counted, and it was observed that the number of *E. coli* dropped sharply to below 10^3^ under the photothermal effect of Bi (Fig. 6D), while the number of *S. aureus* dropped to below 500 CFU/ml (Fig. 6F). Bacteria plate culture images could be clearly observed, where under the same dilution factor the number of colonies in the GO-PS/Bi and GO-PS/HAL@Bi hydrogel treatment groups irradiated by NIR laser was significantly lower than that of the other 3 groups, confirming that the photothermal effect also had a good bactericidal effect in vivo.

### Quality evaluation of GO-PS/HAL@Bi hydrogel in promoting wound healing

**Fig. 7.**
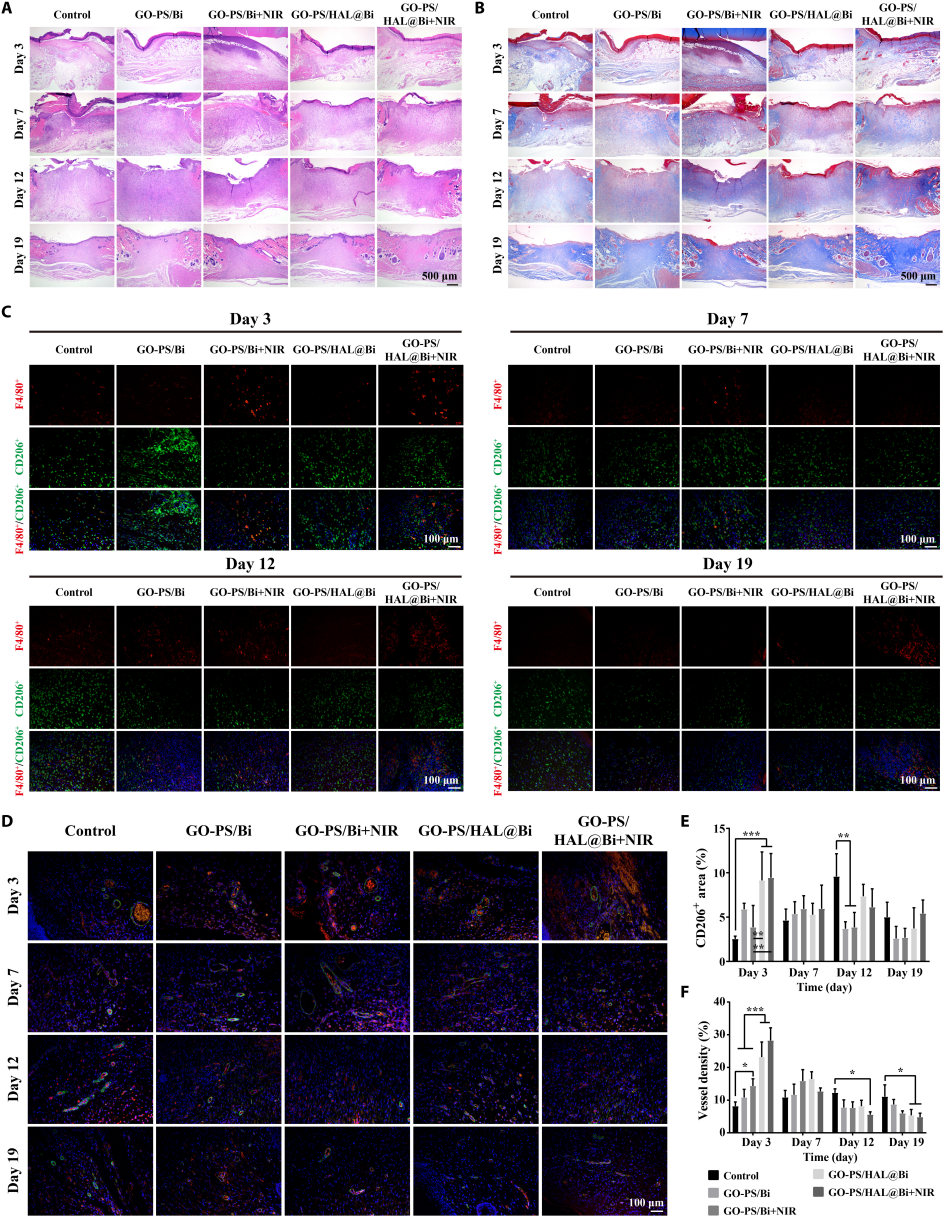
Histological evaluation of wound healing quality. (A) H&E staining and (B) Masson staining images of skin on 3, 7, 12, and 19 d. Scale bar, 500 μm. (C) Immunofluorescence staining images of F4/80 and CD206 of skin on 3, 7, 12, and 19 d. Scale bar, 100 μm. (D) Immunofluorescence staining images of CD31 (red) and α-SMA (green) of skin on 3, 7, 12, and 19 d. Scale bar, 100 μm. (E) Statistical value of CD206^+^ area (*n* = 4, *P < 0.05, **P < 0.01, ***P < 0.001). (F) Statistical value of vessel density calculated from immunofluorescence staining images (*n* = 4, *P < 0.05, **P < 0.01, ***P < 0.001).

In order to compare the quality of wound healing, hematoxylin and eosin (H&E) and Masson staining were performed on wound tissue at different healing stages to compare the role of the Bi-containing hydrogel in the wound healing process from a histological perspective. The results of H&E staining showed that a large number of dark purple mononuclear cells gathered at the wound site within the first 7 d because of the severe inflammatory reaction at the wound site due to bacterial infection. After 7 d of treatment, the granulation tissue gradually thickened. The tissues of the GO-PS/Bi + NIR, GO-PS/HAL@Bi, and GO-PS/HAL@Bi + NIR treatment groups gradually remodeled after 12 d, and the capillaries in the granulation tissue gradually shrank. The number of fibroblasts and macrophages that proliferated in the early stage gradually decreased, and the collagen secreted by fibroblasts gradually filled the injury site. The wound was completely closed after 19 d of treatment (Fig. 7A and B).

Histopathological analysis via H&E staining after 3 d of treatment revealed no significant differences in the morphology of normal skin tissue at the injury boundary between the GO-PS/HAL@Bi + NIR group and the GO-PS/HAL@Bi group. No characteristic features of burns or inflammation, such as epidermal necrosis, dermal degeneration, or vascular dilation, were observed. Despite the fact that during treatment NIR irradiation raised the local temperature to 50.8 °C, which was intended to reduce bacterial load at the infected wound site, no significant thermal damage or inflammatory response was detected in the surrounding normal skin tissue. This suggests that, under our experimental conditions, the photothermal effect of NIR did not induce adverse effects on the healthy skin adjacent to the wound (Fig. 7A).

The macrophages at the wound site were further marked, and the number of M2 macrophages that increased in the first 12 d decreased after the wound was healed (day 19). On day 3, the number of CD206^+^ cells in the hydrogel treatment group with HAL was more, indicating that HAL can participate in the regulation of M2 polarization of macrophages (Fig. 7C). After quantitative statistics of the fluorescent area of CD206^+^ cells, we found that the generation of M2 macrophages would be advanced to the 3rd day with the participation of HAL, thereby accelerating the process of tissue regeneration (Fig. 7E). We observed that Bi could promote vascularization in cell experiments, so blood vessels were labeled in skin tissue to further verify the physiological role of Bi. The blood vessel density of the hydrogel containing Bi group was higher than that of the control group, and a large number of blood vessels began to form after 3 d of treatment and gradually shrank after the 12th day. The vascular density in the control group was low in the early stage, and a large number of blood vessels still remained after 19 d of treatment, indicating that the remodeling stage had not been completed (Fig. 7D and F).

In general, the hydrogel containing Bi promoted the formation of blood vessels and accelerate the production of cells to quickly form granulation tissue to fill the injured area, as the results obtained in vitro. After loading HAL, the GO-PS/HAL@Bi hydrogel also promoted the formation of M2 macrophages and increased the speed of the skin regeneration process.

## Conclusion

For the first time, Bi was found to promote migration of HaCaT cells and tube formation of HUVECs. The hollow Bi nanoparticles were used as drug carriers to deliver HAL to promote the M2 polarization of macrophages. Gelatin-based biomaterials modified by methacrylation and ODex form double crosslinked hydrogels. The material itself and the loaded HAL@Bi can play a certain role in regulating the phenotype of macrophages. The GO-PS/HAL@Bi hydrogel was verified as a promising material for wound repair in both cells and rats, promoting vascularization and M2 macrophage polarization and achieving antibacterial effects through the photothermal effect. However, the existing researches related to tissue repair seldom explore the biological effects of pure Bi. While we have observed some repair effects, the repair mechanism has not been thoroughly investigated. It is expected that more researchers will be inspired by this study to apply Bi element or its preparations to tissue repair and to develop new biomedical application for Bi in the future.

## Data Availability

The data that support the findings of this study are available from the corresponding author upon reasonable request.
