# Supplementary material for: Hollow Bismuth Nanoparticle-Loaded Gelatin Hydrogel Regulates M2 Polarization of Macrophages to Promote Infected Wound Healing
Source: Biomater Res. 2024 Nov 11;28:0105. doi: 10.34133/bmr.0105 (PMC11551490; doi:10.34133/bmr.0105)
Supplement: Supplementary 1 — Figs. S1 to S6 [file bmr.0105.f1.docx]

**Supporting information**

**Hollow Bismuth Nanoparticles-loaded Gelatin Hydrogel Regulates M2 Polarization of Macrophages to Promote Infected Wound Healing**

Dongming Lv^1, #^, Zhongye Xu^1, #^, Hao Yang ^1, #^, Yanchao Rong^1^, Zirui Zhao^1^, Zhicheng Hu^1^, Rong Yin^2^, Rui Guo^3, *^, Xiaoling Cao^1, *^, Bing Tang^1, *^

^1^Department of Burns, Wound Repair and Reconstruction, the First Affiliated Hospital of Sun Yat-sen University, Guangzhou 510080, Guangdong, China.

^2^Department of Dermatology, the First Affiliated Hospital of Sun Yat-sen University, Guangzhou 510080, Guangdong, China.

^3^ Key Laboratory of Biomaterials of Guangdong Higher Education Institutes, Key Laboratory of Regenerative Medicine of Ministry of Education, Guangdong Provincial Engineering and Technological Research Center for Drug Carrier Development, Department of Biomedical Engineering, Jinan University, Guangzhou 510632, Guangdong, China.

^#^These authors contributed equally

*Correspondence: [guorui@jnu.edu.cn](mailto:guorui@jnu.edu.cn) (Rui Guo), caoxling3@mail.sysu.edu.cn (Xiaoling Cao), [tangbing@mail.sysu.edu.cn](mailto:tangbing@mail.sysu.edu.cn) (Bing Tang)


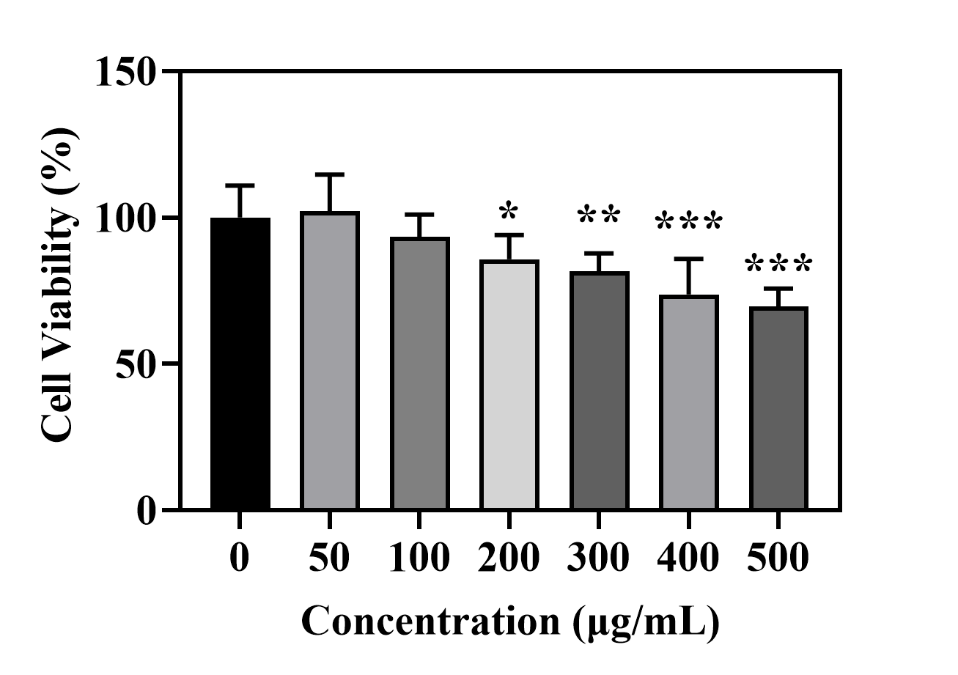


**Figure S1.** Cell viability of HUVEC cells incubation with different concentration of hollow bismuth nanoparticles for 24 h.

**
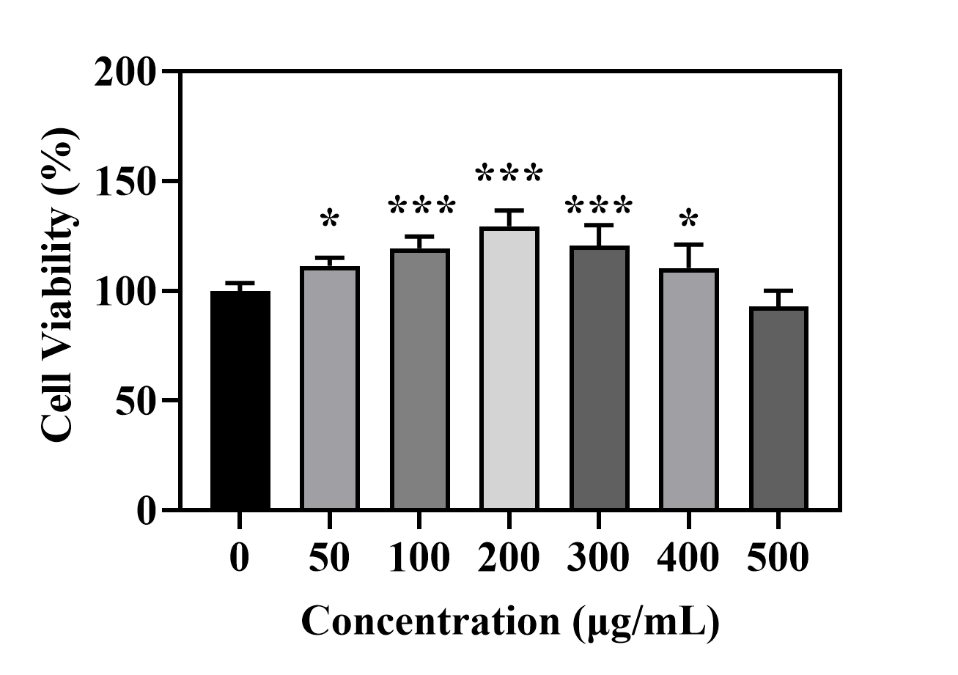
**

**Figure S2.** Cell viability of HaCaT cells incubation with different concentration of hollow bismuth nanoparticles for 24 h.


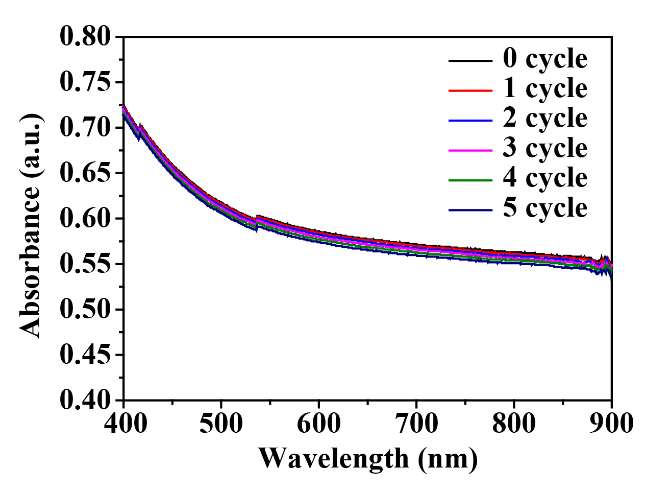


**Figure S3.** UV-Vis spectra of hollow Bi (200 μg/mL) without irradiation on 5 cycles.


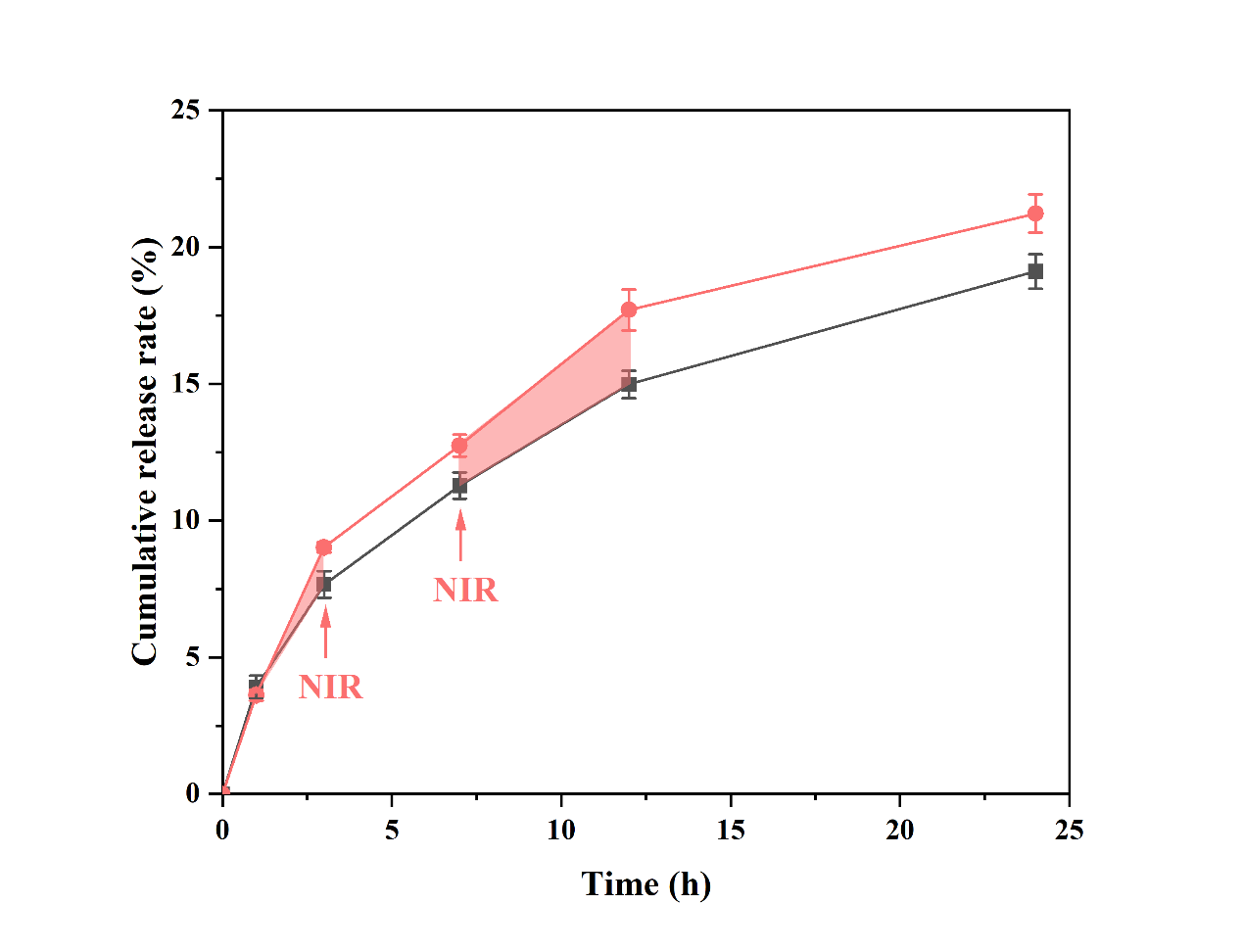


**Figure S4.** HAL release from HAL@Bi under the NIR laser trigger.


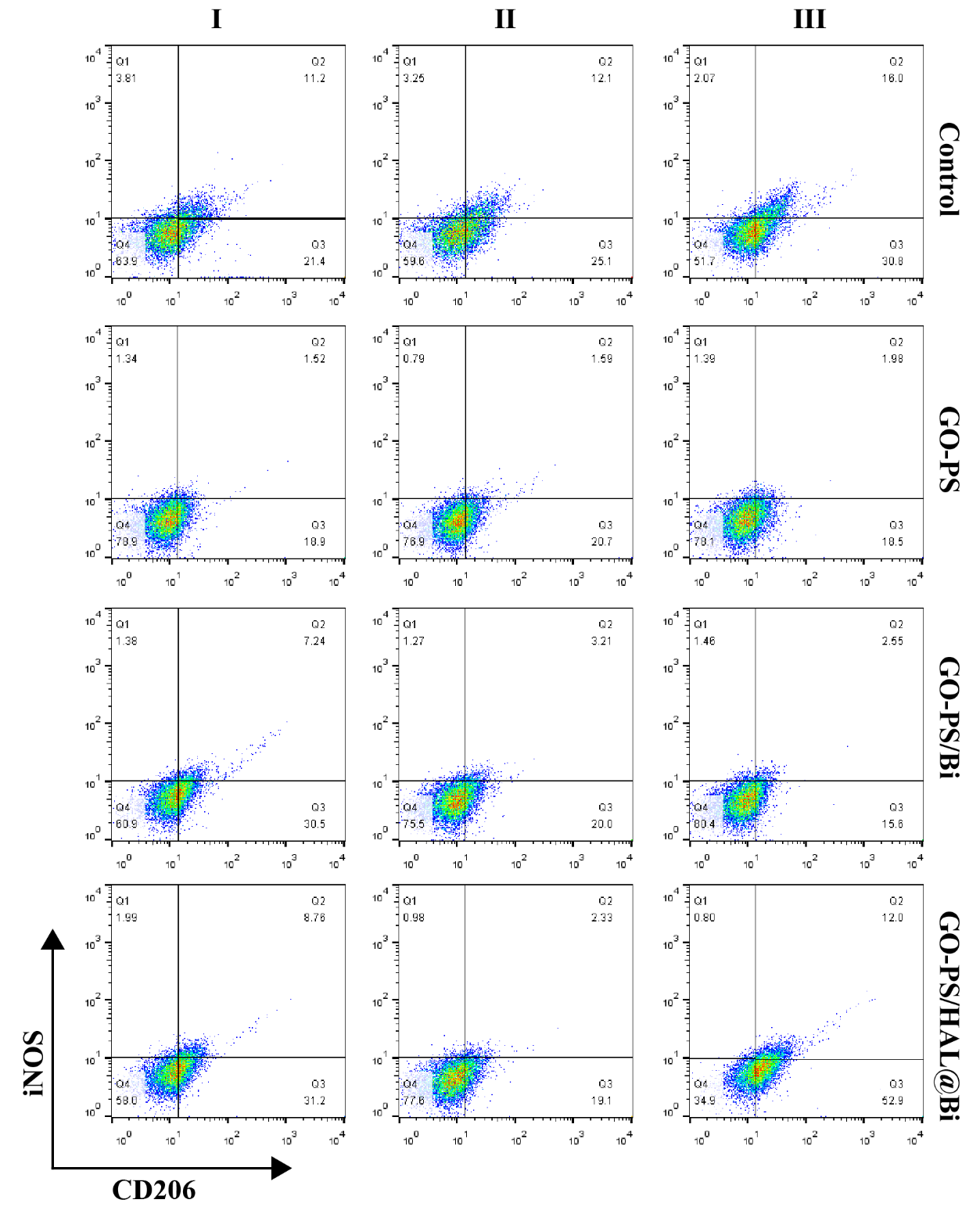


**Figure S5.** Images of flow cytometry analysis of LPS induced RAW264.7 cells treated with GO-PS, GO-PS/Bi, or GO-PS/HAL@Bi.

**
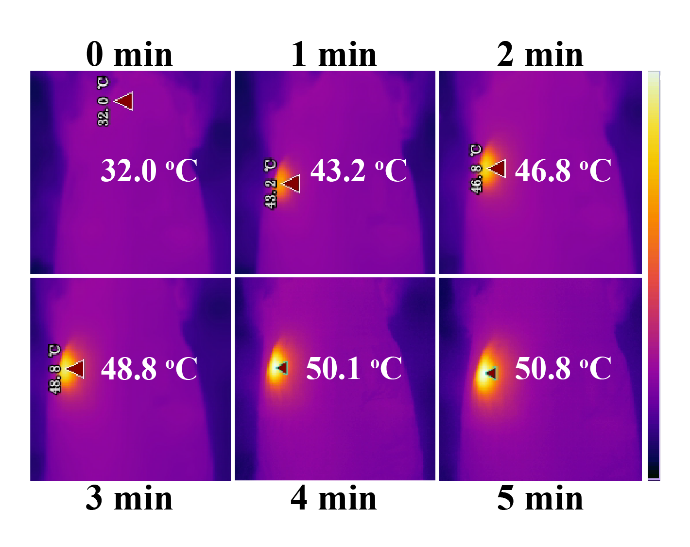
**

**Figure S6.** Thermal image of GO-PS/HAL@Bi hydrogel on SD rat wound surface after near-infrared light irradiation.
